# Tailoring the Structural and Optical Properties of Germanium Telluride Phase-Change Materials by Indium Incorporation

**DOI:** 10.3390/nano11113029

**Published:** 2021-11-12

**Authors:** Xudong Wang, Xueyang Shen, Suyang Sun, Wei Zhang

**Affiliations:** 1 Center for Alloy Innovation and Design (CAID), State Key Laboratory for Mechanical Behavior of Materials, Xi’an Jiaotong University, Xi’an 710049, China; xudong.wang@stu.xjtu.edu.cn (X.W.); v32267209@stu.xjtu.edu.cn (X.S.); sy.sun@stu.xjtu.edu.cn (S.S.); 2 Pazhou Lab, Pengcheng National Laboratory in Guangzhou, Guangzhou 510320, China

**Keywords:** phase change materials, amorphous phase, germanium telluride, indium alloying, optical contrast

## Abstract

Chalcogenide phase-change materials (PCMs) based random access memory (PCRAM) enter the global memory market as storage-class memory (SCM), holding great promise for future neuro-inspired computing and non-volatile photonic applications. The thermal stability of the amorphous phase of PCMs is a demanding property requiring further improvement. In this work, we focus on indium, an alloying ingredient extensively exploited in PCMs. Starting from the prototype GeTe alloy, we incorporated indium to form three typical compositions along the InTe-GeTe tie line: InGe_3_Te_4_, InGeTe_2_ and In_3_GeTe_4_. The evolution of structural details, and the optical properties of the three In-Ge-Te alloys in amorphous and crystalline form, was thoroughly analyzed via ab initio calculations. This study proposes a chemical composition possessing both improved thermal stability and sizable optical contrast for PCM-based non-volatile photonic applications.

## 1. Introduction

Non-volatile memory (NVM) is a rising technology that allows for high-density data storage and fast data processing [1,2,3,4,5,6]. Phase-change materials (PCMs)-based random access memory (PCRAM) is a leading NVM candidate with successful stand-alone memory products such as Intel Optane. By improving its thermal stability, PCRAM is also a promising candidate for embedded memory [7,8]. As announced by STMicroelectronics, PCRAM will be used as embedded memory, replacing Flash memory, for their future microcontroller units (MCU) for the automotive industry [7]. Moreover, PCRAM is also being exploited for more advanced applications, including neuro-inspired computing [9,10,11,12,13,14,15], stochasticity-based computing [16,17], flexible electronics [18], optical displays [19,20,21], all-optical computers [22,23,24,25,26], low-loss optical modulators [27], metasurfaces [28,29,30,31,32] and others [33,34].

PCMs can be switched rapidly and reversibly between their amorphous and crystalline phases via Joule heating induced by electrical or optical pulses [1,35]. The notable contrast in either electrical resistivity or optical reflectivity between each phase is utilized to encode digital information [1]. Several demanding requirements, such as high programming speed, good thermal stability, low power consumption, stable property contrast window and long cycling endurance, have to be well satisfied for high-performance PCRAM. Germanium chalcogenides, in particular, GeTe and GeTe-Sb_2_Te_3_ pseudo-binary compounds (GST), especially Ge_2_Sb_2_Te_5_ [36], are one of the most successful material families that could meet these challenging requirements simultaneously. Doping and alloying are frequently used to tailor the material properties for faster speed and/or better retention temperature, targeting different application scenarios [37,38,39,40,41,42,43,44].

For decades, indium has been an important alloying element used in rewritable optical data storage products [45]. The flagship PCM is AgInSbTe [45,46,47,48]. Recently, indium-alloyed GeTe [49,50,51,52] and GST [53,54] were reported, and their enhanced amorphous stability makes them suitable candidates for high-temperature PCRAM applications. In addition, indium forms a unique PCM In_3_SbTe_2_ [55,56,57,58,59] that exhibits metallic behavior in its crystalline phase, but semiconducting behaviors in its amorphous phase, in contrast to conventional PCMs, which remain semiconducting during memory programming. It has been suggested that even InTe could also be a potential PCM for non-volatile electronics [53,60]. In this work, we focus on the InTe-GeTe (IGT) tie line, in particular, the three stoichiometric compositions, namely InGe_3_Te_4_, InGeTe_2_ and In_3_GeTe_4_. By performing thorough ab initio calculations and chemical bonding analyses, we elucidate the role of indium in altering the structural and optical properties of GeTe.

## 2. Computational Details

We performed ab initio molecular dynamics (AIMD) simulations based on density functional theory (DFT) to generate melt-quenched amorphous structures [61]. The second-generation Car-Parrinello method [62] as implemented in CP2K package [63] was employed along with Perdew–Burke–Ernzerhof (PBE) functional [64] and the Goedecker pseudopotentials [65]. The canonical NVT ensemble was used and the time step was set at 2 fs. Vienna Ab-initio Simulation Package (VASP) [66] was employed to relax the amorphous structures and crystalline counterparts, prior to the calculations of electronic structure and optical response. For VASP calculations, we applied the PBE functional and projector augmented-wave (PAW) pseudopotentials [67]. The energy cutoff for plane waves was set at 500 eV. Chemical bonding analyses were conducted with the LOBSTER code [68,69,70]. Crystal orbital Hamilton populations (COHP) were applied to separate the covalent interactions into bonding (positive −COHP) and antibonding (negative −COHP) contributions. Bader charges were calculated to evaluate the atomic charge transfer in the structures [71]. Frequency-dependent dielectric matrix was calculated within the independent-particle approximation without considering local field effects and many body effects, which proved to be adequate to account for the optical contrast between crystalline and amorphous PCMs [72,73,74]. The absorption α(ω) and reflectivity R(ω) can be calculated from the dielectric functions [75]:

(1) $$\alpha \left(\omega \right)=\frac{\sqrt{2}\omega }{c}\;{\left(\sqrt{\varepsilon_{1}^{2}+\varepsilon_{2}^{2}}-\varepsilon_{1}\right)}^{\frac{1}{2}}$$

(2) $$R\left(\omega \right)=\frac{{\left(n-1\right)}^{2}+k^{2}}{{\left(n+1\right)}^{2}+k^{2}}$$

where $\varepsilon_{1}$ and $\varepsilon_{2}$ are the real and imaginary parts of the dielectric function. n and k are the refractive index and extinction coefficient, which can be calculated from the dielectric functions:

(3) $$n\left(\omega \right)={\left(\frac{\sqrt{\varepsilon_{1}^{2}+\varepsilon_{2}^{2}}+\varepsilon_{1}}{2}\right)}^{\frac{1}{2}}\;$$

(4) $$k\left(\omega \right)={\left(\frac{\sqrt{\varepsilon_{1}^{2}+\varepsilon_{2}^{2}}-\varepsilon_{1}}{2}\right)}^{\frac{1}{2}}$$

Van der Waals correction based on the Grimme’s D3 method was considered in all AIMD and DFT calculations [76,77]. All the electronic structures, chemical bonding and optical properties were calculated using relaxed structures at zero K with VASP. For standard calculations, only gamma point was used to sample the Brillouin zone of the supercell models, while a 3 × 3 × 3 *k*-point mesh was used to converge the optical response calculations. For statistics, we built three crystalline and three amorphous models for each composition.

## 3. Results and Discussion

**Figure 1:**
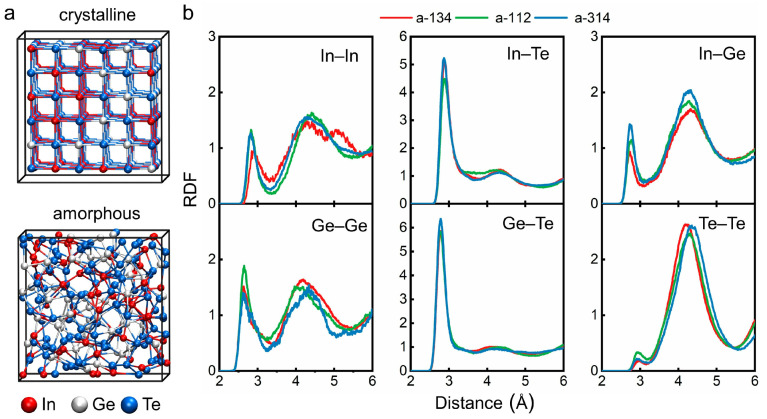
(**a**) Atomic structures for crystalline and amorphous InGeTe_2_. Red, silver and blue spheres represent In, Ge and Te atoms, respectively. (**b**) Partial radial distribution functions (RDFs) for each atomic pair in the three amorphous compounds. The “a-134”, “a-112” and “a-314” represent amorphous InGe_3_Te_4_, InGeTe_2_ and In_3_GeTe_4_, respectively.

As reported in Ref. [78], a single-phase rock-salt structure was obtained over a wide compositional range of 8–75 mole% InTe in IGT at ambient conditions, in which Te atoms occupied one sublattice, while Ge and In atoms shared the other one. The three IGT compositions considered in this work, namely InGe_3_Te_4_, InGeTe_2_ and In_3_GeTe_4_, fall in this compositional range, and were expected to take the rock-salt structure. To account for the compositional disorder of Ge and In atoms on the cation-like sublattice, we built 3 × 3 × 3 supercells (216 atoms in total) and distributed Ge and In atoms using a quasi-random number generator. Three independent models were considered for each composition. Each supercell model was fully relaxed with respect to both atomic coordinates and cell volume by DFT calculations. The relaxed cell-edge of crystalline (c-) InGe_3_Te_4_, InGeTe_2_ and In_3_GeTe_4_ is 18.21, 18.28 and 18.55 Å, respectively. The corresponding unit cell lattice parameters, 6.07, 6.09 and 6.18 Å, are in good agreement with experimental values (5.97, 6.00 and 6.06 Å) [78]. The relaxed structure of c-InGeTe_2_ is shown in Figure 1a and the structures of the other two compositions are shown in Figure S1.

The relaxed crystalline supercells were then used to generate amorphous (a-) models following a melt–quench protocol [61]. The supercell models were quickly heated to a very high temperature to remove the crystalline order. After randomization at 3000 K for 15 ps, the models were quenched down to and equilibrated at 1200 K, above the melting point of IGT alloys (∼550–750 °C) [49] for 30 ps. Amorphous models were then generated by quenching the liquids down to 300 K with a cooling rate of 12.5 K/ps. During this quenching process, we stopped the simulation after every 100 K, and the simulation box size was increased to reduce the internal stress. Within each temperature window, one NVT calculation was performed using a fixed box size. The model was equilibrated at 300 K for 30 ps. This density value was then used to generate two additional melt-quenched amorphous models for each composition. All three amorphous models showed consistently low pressure values below 3 kbar. The obtained cell edges of amorphous InGe_3_Te_4_, InGeTe_2_ and In_3_GeTe_4_ are 18.90, 19.08 and 19.32 Å, respectively. This increase in the cell edge of the amorphous phase is consistent with the trend observed in their crystalline counterparts. Further optimization of the internal stress or the use of the NPT ensemble for melt-quench simulations could potentially lead to some numerical differences in the mass density, but is not expected to alter the amorphous structures much.

The amorphous structure of InGeTe_2_ is shown in Figure 1a and the snapshots of the other two compositions are in Figure S1. The partial radial distribution functions (RDFs) of each atomic pair in the three amorphous compounds are shown in Figure 1b. The peak positions of the heteropolar bonds In–Te (2.87 Å) and Ge–Te (2.78 Å) are not varied with composition, whereas the homopolar or “wrong” bonds show small shifts. As developed in our previous work [79], the “bond-weighted distribution function (BWDF)” provides direct information on the length of chemical bonds in amorphous IGT alloys (Figure S2). Despite the change in chemical composition, the bond length shows very similar values in amorphous IGT alloys, i.e., Ge–Te 3.20 Å, In–Te 3.40 Å, Ge–Ge 3.20 Å, In–In 3.25 Å and Ge–In 3.40 Å. In all three amorphous IGT alloys, Te–Te shows mostly antibonding interactions. These bond length values are used as cutoffs for the interatomic distance for the following structural analysis.

**Figure 2:**
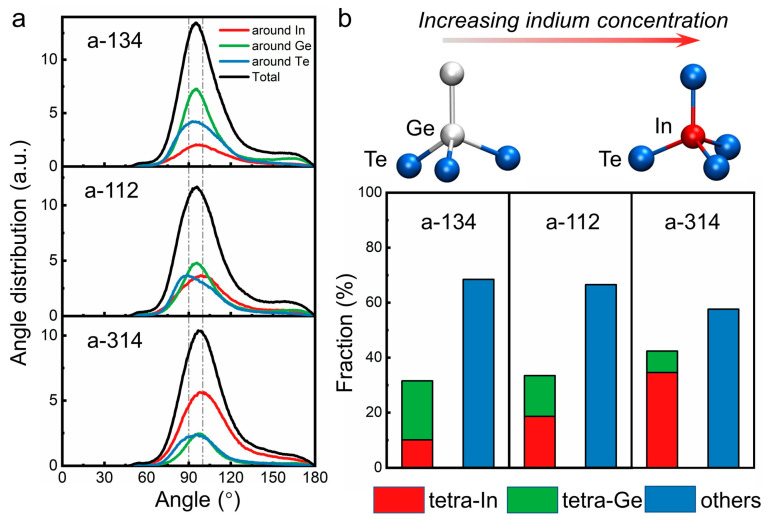
(**a**) Angle distribution functions (ADFs) for the amorphous structures of the three In-Ge-Te compositions. The central bond angles for perfect tetrahedron (109.5°) and octahedron (90°) are marked as dashed lines in ADF plots. (**b**) Ratios of tetrahedral motifs in the three amorphous structures. Typical Ge- and In-centered tetrahedral motifs (short as tetra-In and tetra-Ge) in the amorphous structures are shown on the top panel.

The angle distribution function (ADF) of the three amorphous structures (Figure 2a) shows that In and Ge atoms mainly form local motifs with the central bond angles ranging from 90° to 109.5°, which corresponds to the bond angles in octahedral and tetrahedral motifs, respectively. As the concentration of indium increases, the ADF peak for indium atoms clearly shifts toward 109.5°, implying an increase in indium-centered tetrahedral motifs. We used bond order parameter *q* [80] to quantify the fraction of tetrahedral motifs in amorphous IGT alloys. Such parameters are frequently used for the structural analysis of amorphous PCMs [81,82,83]. As shown in Figure 2b, as indium concentration increases, the total fraction of In- and Ge-centered tetrahedral motifs (short as tetra-In and tetra-Ge) increases from 31.6% (a-InGe_3_Te_4_), 33.5% (a-InGeTe_2_) to 42.4% (a-In_3_GeTe_4_) in relation to the total number of In and Ge atoms. Specifically, the fraction of tetra-In increases from 10.1%, 18.6% to 34.6%, while the fraction of tetra-Ge decreases from 21.5%, 14.9% to 7.8%. The fraction of tetrahedrons for a-InGeTe_2_ is smaller than that reported in previous work (38 % in total, with 31.5% for tetra-In and 6.5% for tetra-Ge) [50], due to the different choice of cutoff values for the interatomic distance and the deviation in calculated density values (vdW interactions were included in the current work). The ratios of tetrahedral motifs in amorphous IGT alloys are all higher than that of their parent phase—GeTe, where 25–30% tetrahedral atoms are typically found in the rapidly quenched amorphous phase [84,85,86].

**Figure 3:**
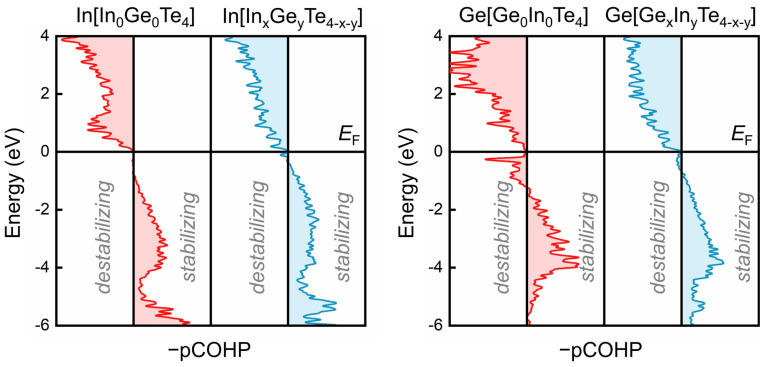
Projected COHP (pCOHP) for tetra-In and tetra-Ge motifs in a-InGeTe_2_. Tetrahedral motifs are classified as the ones with only heteropolar bonds, denoted as In[In_0_Ge_0_Te_4_] and Ge[Ge_0_In_0_Te_4_], and the others with at least one wrong bond indicated as In[In_x_Ge_y_Te_4−x−y_] and Ge[Ge_x_In_y_Te_4−x−y_] (x or y ≥ 1, x + y ≤ 4).

In addition to the increase in the total number of tetrahedral motifs from a-InGe_3_Te_4_ to a-In_3_GeTe_4_, the local bonding configuration also shows a major difference. Despite the change in chemical compositions, nearly all the tetra-Ge atoms are bonded with at least one Ge or In atom, while the majority of tetra-In atoms are heteropolar-bonded in the three amorphous IGT alloys. To quantify the role of “wrong” bonds, we carried out a projected COHP (pCOHP) analysis. As shown in Figure 3 and Figure S3, the pCOHP of heteropolar-bonded tetra-Ge atoms demonstrates sizable antibonding interactions right below Fermi energy (*E*_F_), and the presence of wrong bonds (including Ge–Ge and Ge–In) largely reduces such antibonding contributions, stabilizing the tetrahedral motifs locally. In contrast, the pCOHP of tetra-In atoms with and without wrong bonds mostly shows bonding interactions below *E*_F_. These results are consistent with the bonding configuration in the two parent phases, a-GeTe [79] and a-InTe [83], though In–Ge bonds are present in all three amorphous IGT alloys. Since indium atoms do not require homopolar bonds to stabilize tetrahedral motifs, the ratio of tetrahedral units is increased with the indium concentration. As compared to their crystalline counterparts, where all In and Ge atoms are octahedrally bonded, the enlarged structural deviation will enhance the thermal stability of amorphous IGT alloys. This observation is consistent with experimental findings, as the crystallization temperature *T*_x_ of doped InGeTe_2_ [49] and undoped InTe thin films [60] is increased to ∼276 and ∼300 °C, respectively, as compared to that of GeTe *T*_x_ ∼190 °C [87].

**Figure 4:**
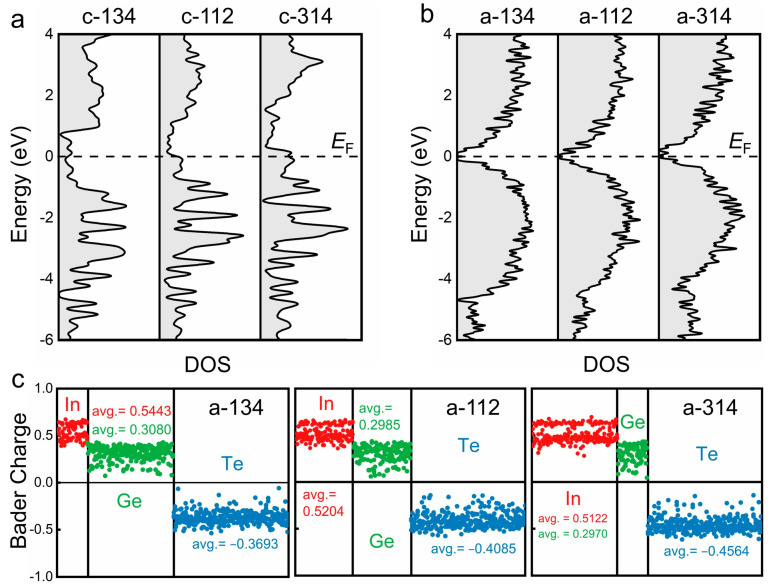
Density of states (DOS) for the (**a**) crystalline and (**b**) amorphous structures of the three In-Ge-Te compositions. The “c-134”, “c-112” and “c-314” represent crystalline InGe_3_Te_4_, InGeTe_2_ and In_3_GeTe_4_, respectively. (**c**) Bader charges (in electrons per atom) of all atoms in the three amorphous structures.

The calculated density of states (DOS) of the three IGT alloys in both crystalline and amorphous forms are shown in Figure 4a,b. Regarding the crystalline models, the overall DOS profiles are quite similar, and all three alloys exhibit metallic features. By contrast, all three amorphous models are narrow-gap semiconductors. Statistical sampling yields consistent results (Figure S4). The large difference in DOS between the crystalline and amorphous IGT results in a wide resistance contrast window for PCRAM applications [49]. The Bader charge analysis (Figure 4c) details larger net charges for In atoms than for Ge atoms due to the difference in electronegativity. The bimodal feature of the charges of indium atoms is consistent with previous work [50], stemming from different local environments of indium atoms. The enlarged charge transfer in amorphous structures increases the probability of long-distance electromigration under the transient electrical field induced by programming pulses [88], which is detrimental to the cycling endurance of devices [89,90]. For RESET operations, the higher the melting temperature *T*_m_, the greater the power consumption. The melting temperature for IGT alloys has a “W” shape profile, according to the InTe-GeTe phase diagram, which shows that InGeTe_2_ has a higher melting temperature *T*_m_ (740 °C) than GeTe and InTe whose *T*_m_ are 715 and 688 °C, respectively [49]. Interestingly, the two other compositions, InGe_3_Te_4_ and In_3_GeTe_4_, demonstrated reduced *T*_m_ (645 and 565 °C). Taking into account all these factors for practical applications, we would suggest keeping the IGT composition within the range of InGeTe_2_ to In_3_GeTe_4_ for balanced device performance.

The enhanced amorphous stability is also useful for non-volatile photonic applications [91], yet the incorporation of indium makes IGT alloys metallic, which could affect the optical contrast between the amorphous and crystalline phase. The significant contrast of ∼30% in the optical reflectivity of PCMs stems from a fundamental change in bonding nature from covalent to metavalent bonding (MVB) upon crystallization [92,93,94,95,96,97,98,99]. However, in comparison with GeTe and GST, which have three *p* electrons per site (a key feature of MVB), InTe has a deficient number of *p* electrons, turning the rock-salt phase from semiconducting to metallic. As a result, MVB in IGT alloys is expected to be weakened.

**Figure 5:**
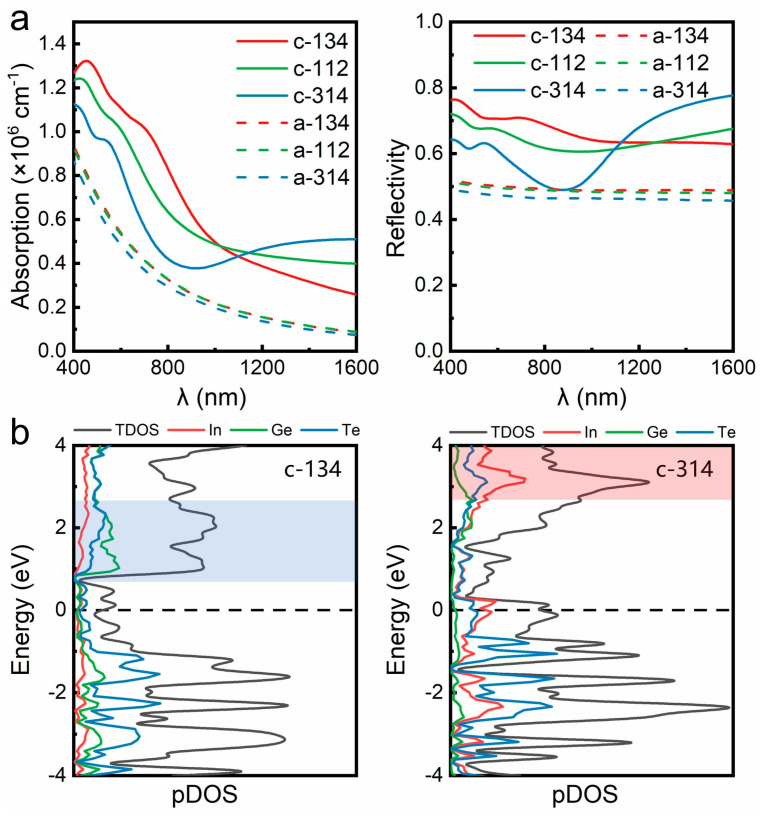
(**a**) Optical absorption and reflectivity of crystalline and amorphous phase of the three In-Ge-Te compositions. (**b**) Projected DOS (pDOS) of c-134 and c-314. Blue and red shaded areas highlight the peak regions of DOS above *E*_F_ in c-134 and c-314, respectively.

For verification, we carried out optical response calculations using the relaxed crystalline and amorphous IGT structures. We focused on the spectrum range from 400 to 1600 nm, covering both the visible light region (∼400 nm to 800 nm) for optical displays [19,20,21] and the telecom wavelength bands (∼1500 to 1600 nm) for silicon-waveguide-integrated photonic applications [23,24,25,26]. As shown in Figure 5a, the optical absorption and reflectivity profiles vary slightly with the chemical compositions in the amorphous phase, while strong changes are found in the crystalline phase. For InGe_3_Te_4_ and InGeTe_2_, sizable contrast in reflectivity between the crystalline and amorphous phases is observed over the whole spectrum, with an average value ∼20%. However, for In_3_GeTe_4_, the contrast in reflectivity nearly vanishes at around ∼900 nm, and it becomes greater below 700 nm, or above 1000 nm.

The three IGT crystals have a crossover at ∼1100 nm. For long wavelength or small photon energy (below ∼1 eV) regions, the optical excitation is mainly determined by states near *E*_F_. With increased DOS near *E*_F_ by heavier indium alloying (Figure 4a), the absorption in the long wavelength region is enhanced. However, for the short wavelength or large photon energy (above ∼2 eV) region, the electronic states of a wider energy range would participate in the optical excitation. According to the projected DOS (Figure 5b), the valence states below *E*_F_ are mainly contributed by Te atoms for all three crystals. However, indium alloying shifts the DOS peak in the conduction band to a higher energy range. Since fewer excited states of low energy could contribute to short wavelength excitation, c-In_3_GeTe_4_ shows the smallest absorption and reflectivity in the short wavelength region (Figure 5a).

We note that our optical calculations were performed using DFT-PBE functional with an independent-particle approximation, excluding local field effects and many body effects. Therefore, the absolute values of optical profiles could vary if more advanced methods are employed. Nevertheless, the observation of weakened optical contrast due to heavier indium alloying should remain valid. Taking into account the enhanced crystallization temperature and the reduced melting temperature, we predict an optimal IGT composition within the ranges of InGeTe_2_ and In_3_GeTe_4_ for high-performance non-volatile photonics. To the best of our knowledge, thorough optical measurements of IGT alloys are still lacking. Therefore, we anticipate future experiments exploring the suitability of IGT alloys for optical and photonic PCM applications.

## 4. Conclusions

In summary, we have carried out systematic ab initio calculations for three typical compositions of indium incorporated GeTe compounds, InGe_3_Te_4_, InGeTe_2_ and In_3_GeTe_4_, to elucidate the evolution of structural and optical properties along the InTe-GeTe tie line. Upon indium alloying, the crystalline phase of all the three alloys turns metallic, while their amorphous counterparts all show semiconducting features with narrow band gaps. This stark contrast in the electronic structure guarantees a large resistance window between amorphous and crystalline In-Ge-Te alloys for electrical PCRAM. Yet, too much indium should be avoided, because the stronger charge transfer could be harmful to cycling endurance due to electromigration. Regarding optical properties, both InGe_3_Te_4_ and InGeTe_2_ show sizable optical contrast between the crystalline and amorphous phases in the spectrum range from 400 nm to 1600 nm, covering both visible-light and telecom bands. Meanwhile, In_3_GeTe_4_ shows a less robust contrast window, due to weakened MVB. Moreover, the increased indium concentration enlarges the ratio of tetrahedral motifs in the amorphous phase and consequently increases the structural barrier for crystallization. The InTe-GeTe phase diagram establishes that the melting temperature reaches minimum around In_3_GeTe_4_, indicating lowest power consumption for melt–quench amorphization. Taking all these factors into account, we suggest that the optimal chemical composition for In-Ge-Te alloys should be located in the range between InGeTe_2_ and In_3_GeTe_4_, which could result in the most balanced device performance for PCM-based non-volatile electronic and photonic applications. Our work should serve as a stimulus for further investigations into indium-incorporated PCMs.

## Data Availability

The data presented in this study are available on request from the corresponding author.

## Supplementary Materials

The following are available online at https://www.mdpi.com/article/10.3390/nano11113029/s1. Figure S1: Atomic structures of crystalline and amorphous InGe_3_Te_4_ and In_3_GeTe_4_ (denoted as c/a-134 and 314); Figure S2: Bond population (left panel) and bond weighted distribution functions BWDFs (right panel) for each interatomic pair in the three amorphous compounds. The crossover values from positive to negative in the BWDFs represent cutoff values for bonding interactions, which are used for structural analysis. Further technical details about BWDF can be found in [79] of the main text; Figure S3: Projected COHP (pCOHP) for tetra-In and tetra-Ge motifs in a-InGe_3_Te_4_ and a-In_3_GeTe_4_. Tetra-hedral motifs are grouped as the ones with only heteropolar bonds, denoted as In[In_0_Ge_0_Te_4_] and Ge[Ge_0_In_0_Te_4_], and the others with at least one wrong bond indicated as In[In_x_Ge_y_Te_4__−x__−y_] and Ge[Ge_x_In_y_Te_4__−x__−y_] (x or y ≥ 1, x + y ≤ 4). In the a-314 structure, Ge[Ge_0_In_0_Te_4_] motif is absent; Figure S4: Density of states (DOS) for crystalline and amorphous structures of the three IGT compositions. Three models for each composition were built, which show consistent results.
