# Supplementary material for: Tailoring the Structural and Optical Properties of Germanium Telluride Phase-Change Materials by Indium Incorporation
Source: Nanomaterials (Basel). 2021 Nov 12;11(11):3029. doi: 10.3390/nano11113029 (PMC8619561; doi:10.3390/nano11113029)
Supplement: Supplementary file 1 [file nanomaterials-11-03029-s001.zip › nanomaterials-1436296-supplementary.pdf]

nanomaterials

MDPI

Supplementary Materials

Tailoring the Structural and Optical Properties of Germanium Telluride Phase-Change Materials by Indium Incorporation

Xudong Wang [1], Xueyang Shen [1], Suyang Sun [1] and Wei Zhang [1,2,*]

[1] Center for Alloy Innovation and Design (CAID), State Key Laboratory for Mechanical Behavior of Materials, Xi'an Jiaotong University, Xi'an 710049, China; xudong.wang@stu.xjtu.edu.cn (X.W.); v32267209@stu.xjtu.edu.cn (X.S.); sy.sun@stu.xjtu.edu.cn (S.S.)
[2] Pazhou Lab, Pengcheng National Laboratory in Guangzhou, Guangzhou 510320, China
* Correspondence: wzhang0@mail.xjtu.edu.cn

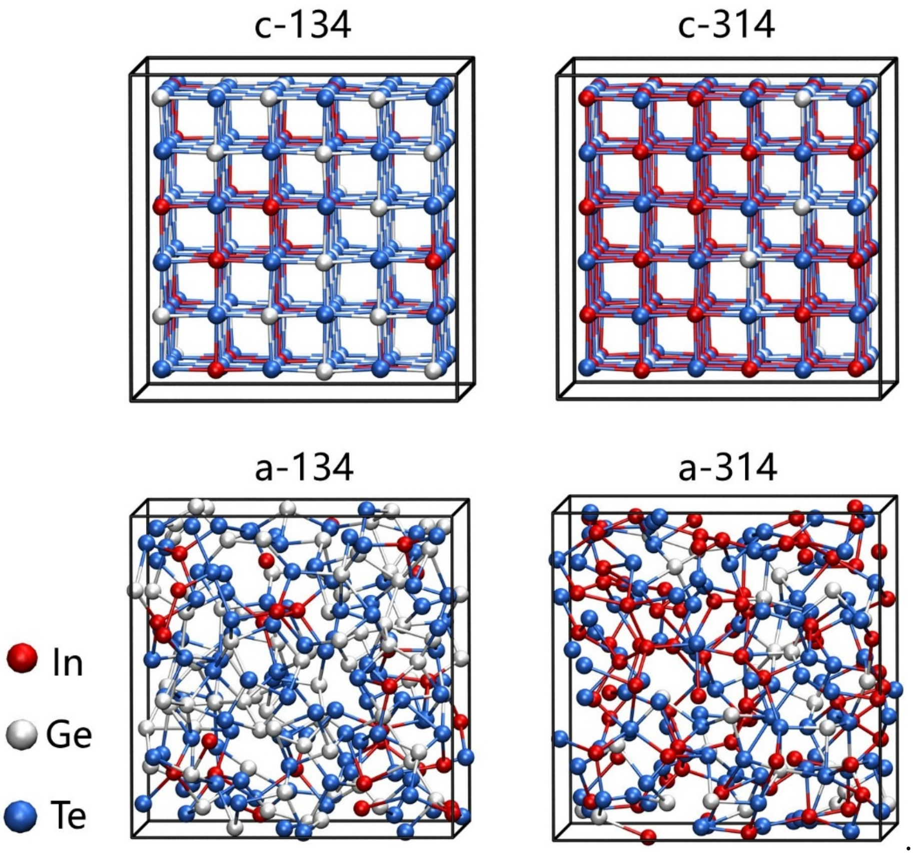

Figure S1. Atomic structures of crystalline and amorphous $InGe_3Te_4$ and In_3GeTe_4 (denoted as c/a-134 and 314).

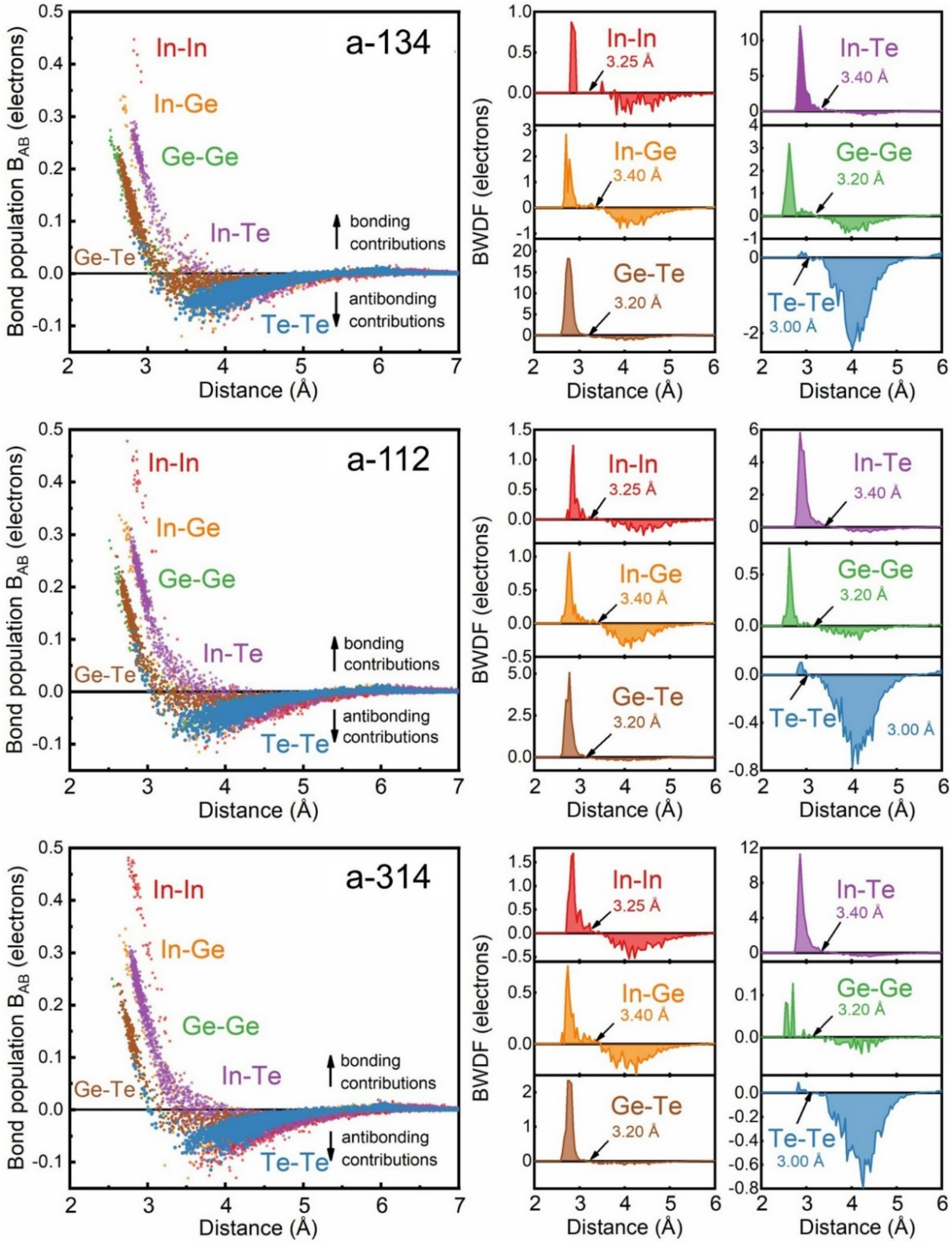

Figure S2. Bond population (left panel) and bond weighted distribution functions BWDFs (right panel) for each interatomic pair in the three amorphous compounds. The crossover values from positive to negative in the BWDFs represent cutoff values for bonding interactions, which are used for structural analysis. Further technical details about BWDF can be found in Ref. 79 of the main text.

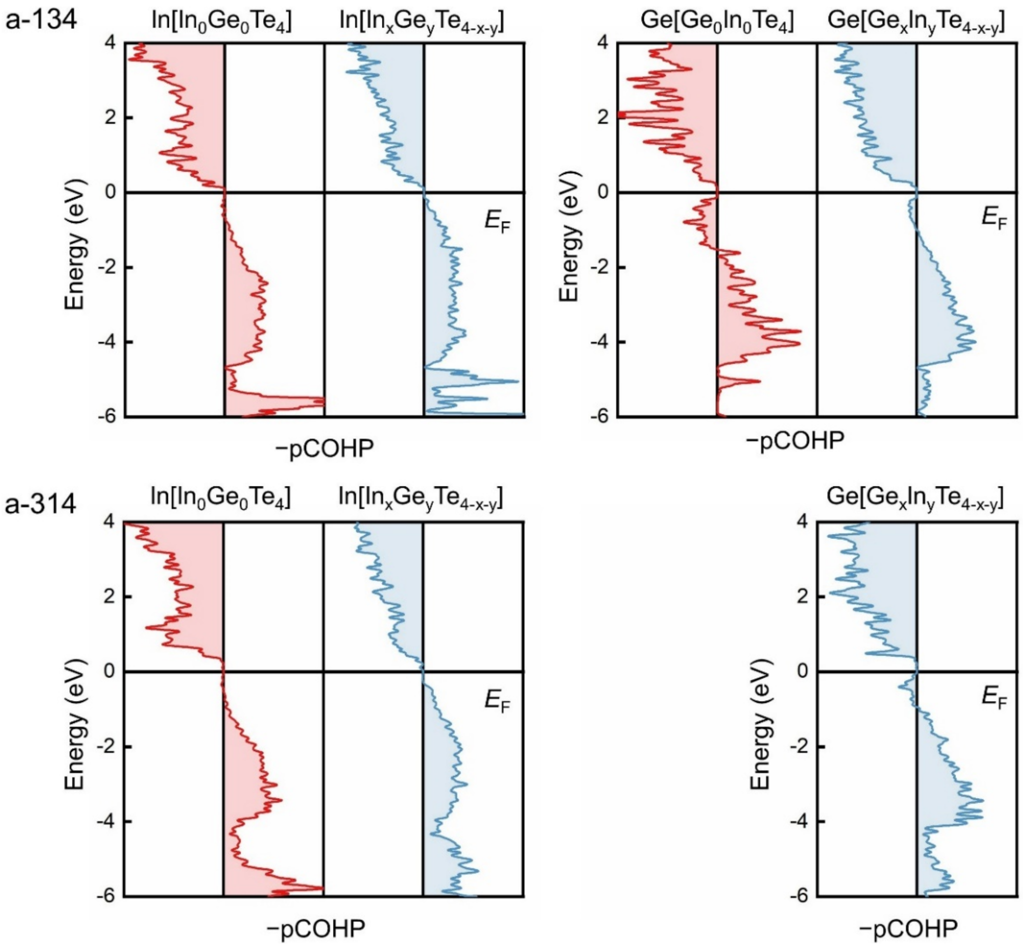

Figure S3. Projected COHP (pCOHP) for tetra-In and tetra-Ge motifs in a-$InGe_3Te_4$ and a-In_3GeTe_4. Tetrahedral motifs are grouped as the ones with only heteropolar bonds, denoted as In[$In_0Ge_0Te_4$] and Ge[$Ge_0In_0Te_4$], and the others with at least one wrong bond indicated as In[$In_xGe_yTe_{4-x-y}$] and Ge[$Ge_xIn_yTe_{4-x-y}$] (x or y ≥1, x+y≤4). In the a-314 structure, Ge[$Ge_0In_0Te_4$] motif is absent.

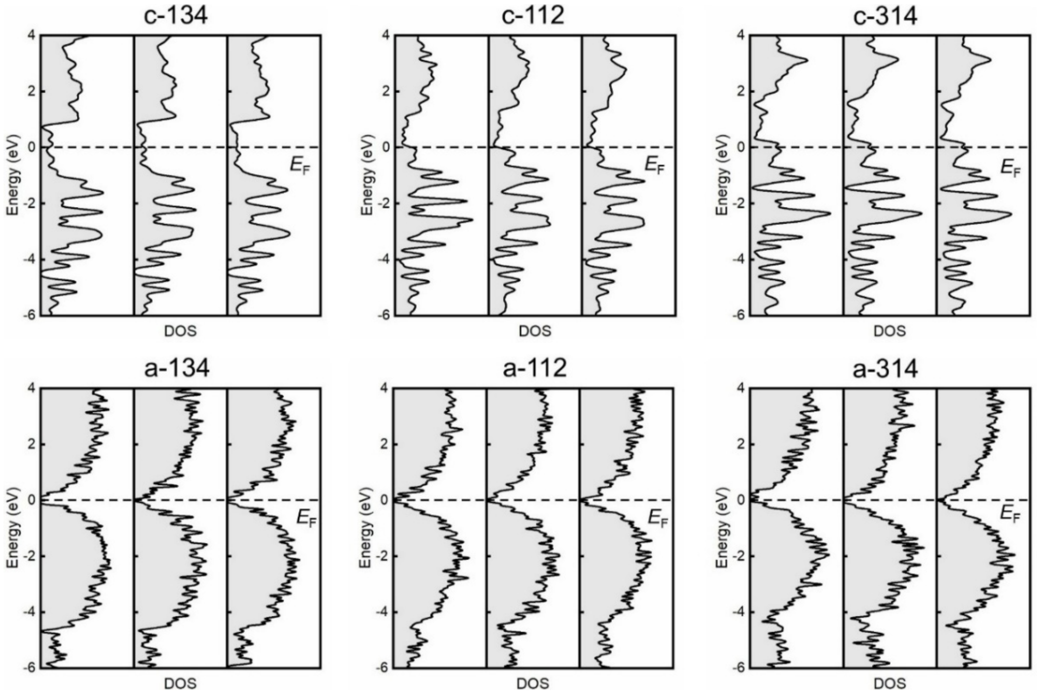

Figure S4. Density of states (DOS) for crystalline and amorphous structures of the three IGT compositions. Three models for each composition were built, which show consistent results.
